# *Trichomonas vaginalis*: a review of epidemiologic, clinical and treatment issues

**DOI:** 10.1186/s12879-015-1055-0

**Published:** 2015-08-05

**Authors:** Patricia Kissinger

**Affiliations:** grid.265219.b0000000122178588School of Public Health and Tropical Medicine, Tulane University, 1440 Canal Street Suite 2004, New Orleans, Louisiana 70112 USA

**Keywords:** *Trichomonas vaginalis*, Trichomoniasis, Epidemiology, Treatment

## Abstract

*Trichomonas vaginalis* (TV) is likely the most common non-viral sexually transmitted infection (STI) in the world. It is as an important source of reproductive morbidity, a facilitator of HIV transmission and acquisition, and thus it is an important public health problem. Despite its importance in human reproductive health and HIV transmission, it is not a reportable disease and surveillance is not generally done. This is problematic since most persons infected with TV are asymptomatic. Metronidazole (MTZ) has been the treatment of choice for women for decades, and single dose has been considered the first line of therapy. However, high rates of retest positive are found among TV infected persons after single dose MTZ treatment. This has not been explained by drug resistance since in vitro resistance is only 2–5 %. Treatment failure can range from 7–10 % and even higher among HIV+ women. Treatment efficacy may be influenced by vaginal ecology. The origins of repeat positives need further explanation and better treatment options are needed.

## Introduction

TV is likely the most common non-viral sexually transmitted infection (STI) in the world. While not a reportable disease, the World Health Organization estimated that there were 276.4 million cases in 2008 and nearly 90 % of these infections occurred among people living in resource-limited settings [1]. TV is more prevalent that *Chlamydia trachomatis, Neisseria gonorrhoeae*, and syphilis combined. The global prevalence of TV has been estimated at 8.1 % for women and 1.0 % for men [2]. These rates may be underestimates as they are derived from studies that used microscopy rather than the more sensitive nucleic acid amplification tests (NAAT) and no formal surveillance systems exist.

With no surveillance programs in place, the epidemiology of TV is not completely known. It is known, however, to vary greatly by population and geography. In the United States, two population-based studies that used PCR testing found rates of 2.3 % among adolescents [3] and 3.1 % among women 14–49 [4]. Population-based studies in Africa show distinctly higher rates. In Zimbabwe the rate was 9.5 % among both genders using antibody testing [5]. Using NAAT, the positivity rate among men in Tanzania was 11 % [6]. Women in Papau New Guinea also appear to have exceptionally high TV rates ranging from 21 % in pregnant women to 42.6 % in the general population [7, 8]. Other population-based studies that used NAAT testing among reproductive aged women in other parts of the world found lower rates (i.e. 1 % in rural Vietnam [9] and 0.37 % in Flanders, Belgium [10], 2.9 % in Shandong Province in China [11].) Screening rates among women attending antenatal or family-planning clinics are often used as an indicator of the prevalence in the general population. Studies at these sites found prevalence rates from 3.2–52 % in resource limited settings and 7.6–12.6 % in the US [12]. Thus, rates of TV vary greatly and are dependent on the risk factor profile of the population.

In general, Africans or persons of African descent have higher rates of TV, as evidenced by higher rates in Sub-Saharan Africa [5, 6], and among persons of African descent such as Garifunas [13] and African Americans in the US [4, 14]. In the United States, the highest prevalence of TV infection in US women is seen among African-Americans with rates ranging from 13–51 % [15]. African American women have rates that are ten times higher than white women, constituting a remarkable health disparity [4]. Other risk factors for TV include increased age, incarceration, intravenous drug use, commercial sex work [16] and the presence of bacteria vaginosis [17].

### Pathogenesis of TV

TV is a flagellated parasitic protozoan, typically pyriform but occasionally amoeboid in shape, extracellular to genitourinary track epithelium with a primarily anerobic lifestyle [18]. The individual organism is 10–20 μ m long and 2–14 μ m wide. Four flagella project from the anterior portion of the cell and one flagellum extends backwards to the middle of the organism, forming an undulating membrane. An axostyle extends from the posterior aspect of the organism. TV has a large genome (strain G3, 176,441,227 bp) with ~ 60,000 protein coding genes organized into six chromosomes [19]. TV is a highly predatory obligate parasite that phagocytoses bacteria, vaginal epithelial cells and erythrocytes and is itself ingested by macrophages. TV uses carbohydrates as its main energy source via fermentative metabolism under aerobic and anaerobic conditions. Incubation time is generally between 4 and 28 days [20].

TV primarily infects the squamous epithelium of the genital tract. TV resides in the female lower genital tract and the male urethra and prostate, where it replicates by binary fission. TV is transmitted among humans, its only known host, primarily by sexual intercourse. Infection may persist for long periods, possibly months or even years, in women but generally persists less than 10 days in males [21]. The parasite does not appear to have a cyst form and does not survive well in the external environment, but can survive outside the human body in a wet environment for more than three hours [22]. There may, however, may be a pseudocyst form. TV pseudocyst have been found to be more virulent in animals and could have relevance for human, particularly in the case of neoplasia [23, 24]. While thought to be rare [20], evidence of non-sexual transmission via fomites and possibly water has been described [25–27]. TV can be infected with double-stranded RNA (dsRNA) viruses that may have important implication for trichomonal virulence and disease pathogensis [28].

### Clinical features of TV

The majority of women (85 %) [4] and men (77 %) [29] with TV are asymptomatic. One third of asymptomatic women become symptomatic within 6 months [20]. Among those who do have symptoms, they include urethral discharge and dysuria. Among women, common sites of infection include the vagina, urethra and endocervix. Symptoms include vaginal discharge (which is often diffuse, malodorous, yellow-green), dysuria, itching, vulvar irritation and abdominal pain. The normal vaginal pH is 4.5, but with TV infection this increases markedly, often to >5 [20]. *Coplitis macularis* or strawberry cervix is seen in about 5 % of women, though with colposcopy this rises to nearly 50 % [30]. Other complications include infection of the adnexa, endometrium, and Skene and Bartholin glands. In men, it can cause epididymitis, prostatitis, and decreased sperm cell motility [31].

### Sequelae of TV

#### Reproductive outcomes

Studies show an association between TV and vaginitis, cervicitis, urethritis, bacterial vaginosis, candidiasis, herpes simplex virus type-1 and type-2, Chlamydia, gonorrhea, and syphilis [32]. TV has also been associated with poor birth outcomes such as low birth weight, preterm delivery, pelvic inflammatory disease, and premature rupture of membranes [33]. One study showed an association between maternal TV infection and intellectual disability in children [34]. Although rare, TV infection can be transmitted perinatally [35] and cause vaginal and respiratory infections in neonates [36, 37].

#### HIV acquisition and transmission

Several cross-sectional and cohort studies that have indicated a higher risk for HIV acquisition among TV+ compared to TV- women [38]. This greater susceptibility is biologically plausible for three reasons: inflammatory response to TV infection results in the increased appearance of HIV target cells [39]; TV infection can impair the mechanical barrier to HIV via punctate mucosal hemorrhages [40]; and TV infection may change the normal vaginal flora rendering it more permissive for bacterial vaginosis [41], which, in turn, can increase the risk of HIV acquisition [42]. These consequences facilitate HIV in TV-infected women. Several studies have also demonstrated increased HIV expression among HIV+/TV+ women. A study by Sorvillo et al. estimates that in a community with a high prevalence of TV, as much as 20 % of HIV could be attributed to TV infection [43]. Chesson et al. estimated that 6.2 % of all HIV infections among US women may be attributable to TV infection [44]. Control of TV, therefore, may provide a cost-effective strategy for reducing HIV transmission especially in settings where TV is common [45, 46] or among subgroups who are at higher risk for TV such as African Americans [47].

Among HIV+ women, TV has been associated with increased HIV vaginal shedding in several studies [38]. Fortunately, treatment for TV has demonstrated reductions in HIV genital shedding in several studies. HIV+ men with urethritis in Malawi, with TV diagnosed by NAAT, experienced a decrease in seminal HIV after MTZ treatment [48]. HIV vaginal shedding was decreased after treatment in one cohort of women, diagnosed by microscopy and culture in Kenya [49], and another, diagnosed by culture, in Louisiana, US [50] These data underscore the importance of screening and treatment among HIV positive persons.

#### HSV-2

TV appears to have a similar bi-directional association with Herpes Simplex virus II (HSV-2) as it does with HIV-1. Concomitant infection with TV has been associated with HSV-2 shedding [51] and women have been found to have TV have a higher incidence of HSV-2 [52].

#### Neoplasia

Evidence that TV is associated with HPV acquisition, thus there may be in indirect link between TV and cervical neoplasia. A meta-analysis found that TV was associated with a 1.9 fold risk of cervical neoplasia [53]. Studies of Finnish, Dutch, Belgian and Chinese women have all found elevated odds (1.4–2.0) of cervical neoplasia among women who have TV or visa versa [10, 54–57]. Sutcliffe et al. found an association between TV and prostate cancer in one study but not in a subsequent study [58, 59].

#### Diagnosis

The diagnosis of TV is becoming more precise and more tests have become available in the last decade. Wet mount microscopy has been used for many decades to diagnose TV. The test is inexpensive, low technology and is point of care, however, it is insensitive, particularly in men. Sensitivities range from 50–70 % depending on the expertise of the reader and should be read within 10 min of collection [60]. While culture has better sensitivity that wet mount, in women it is more expensive, time consuming, and also demonstrates poor sensitivity in men. The lack of sensitivity of culture has been found in longitudinal studies of TV treatment. One study of HIV- and one study of HIV+ women found that that after single dose MTZ treatment, TV infection was non-detectable for months via culture and then reappeared in the absence of reported sexual exposure [61, 62] underscoring the need for more sensitive testing than culture.

Nucleic acid probe techniques are the most sensitive tests, are moderately priced and fast, but require instrumentation. These tests are not considered point-of-care. The APTIMA Trichomonas vaginalis Assay (Hologic Gen-Probe, San Diego, CA) was United States Federal Drug Adminstration (FDA)-cleared in 2011 for use with urine, endocervical and vaginal swabs, and endocervical specimens collected in the Hologic PreserveCyt solution (ThinPrep) from females only. Sensitivity is 95–100 % and specificity is also 95–100 % [63]

There are two point-of-care (POC) tests that have been approved by the U.S FDA for diagnosis of T vaginalis among women, OSOM Trichomonas Rapid Test (Genzyme Diagnostics; Cambridge, MA), an immunochromatographic capillary flow dipstick technology [64] and Affirm VP III (Becton, Dickinson & Co.; Franklin Lakes, NJ), a nucleic acid probe test that evaluates for *TV*, *G. vaginalis*, and *C. albicans* [65]. Both tests are performed on vaginal secretions and have a sensitivity of more than 83 % and a specificity of more than 97 %. Results of the OSOM test are available in about 10 min, while results of the Affirm VP III test can be available within 45 min. Xpert® TV by Cepheid (Sunnyvale, CA) has not been FDA approved but holds promise in resource poor countries and for POC diagnostics in men.

It has been generally thought that only vaginal specimens should be collected for TV testing among women. There is, however, some evidence that endocervical specimens are suitable. Endocervical specimens have been found to be 88 % sensitive and 99 % specific for TV by PCR compared to 90 % and 99 % for vaginal swab [66]. Huppert showed that endocervical specimens were 100 % sensitive and 98 % specific by TMA compared to 100 % sensitivity and specificity for vaginal specimen using latent class analysis [67].

NAAT testing too soon after treatment can result in detection of remnant trichomonad DNA, thus producing false positives. By 2–3 weeks post treatment most remnant DNA has cleared [68], however, one study found a 15 % false positive rate at 3 weeks [69]. The validity of NAAT testing post-treatment needs further examination.

### Management and treatment

#### Treatment with 5-nitroimidazoles

For nearly four decades, metronidazole (MTZ) has been the treatment of choice for TV [70]. MTZ belongs to the 5-nitroimidazole drug family and is reported to have about a 95 % success rate in curing TV along with its related compounds such as tinidazole (TNZ) and seconidazole [71]. The World Health Organization (WHO) and the United States Centers for Disease Control and Prevention (CDC) guidelines for treatment of TV include: MTZ or TNZ 2 gm single dose as the recommended regimens, and MTZ 400–500 mg BID 7 day dose as the alternative treatment regimen. Abstinence from alcohol use should continue for 24 h after completion of MTZ or 72 h after completion of TNZ. If a patient fails single dose MTZ therapy they can be given single dose TNZ or 7 day dose MTZ. If this fails, 2 g MTZ or TNZ for 5 days can be administered. If this fails and there is no history of sexual re-exposure, a consultation for medication resistance testing should be done. Consultation and TV susceptibility testing is available in the U.S. from CDC (telephone: 404-718-4141; website: http://www.cdc.gov/std).

#### Treatment among pregnant and lactation women

MTZ is a class B drug and several meta-analyses have found it to be safe in pregnant women in all stages of pregnancy [72, 73]. TNZ has not been evaluated in pregnant women and remains a class C drug. Treatment with 2 g MTZ is recommended by CDC at any time during pregnancy [74] whereas WHO does not recommend treatment in the first trimester unless it is indicated for prevention of untoward birth outcomes. Both entities suggest 2 g dose.

In lactating women who are administered MTZ, withholding breastfeeding during treatment and for 12–24 h after the last dose will reduce the exposure of the infant to metronidazole. For women treated with TNZ, interruption of breastfeeding is recommended during treatment and for 3 days after the last dose.

#### Treatment of recalcitrant TV or allergies to MTZ/NTZ

Persistent TV is usually treated with multi-dose MTZ or TNZ. The most common reactions reported from metronidazole are urticaria and facial edema, while other adverse reactions include flushing, fever and anaphylactic shock from immediate-type hypersensitivity have been reported. De-sensitization can be done, but only has about a 42 % cure rate [75]. If TV remains persistent or the patient is allergic to these medications, other intravaginal treatments have been studied or are under investigation TV including: Acetarsol [76], Boric acid [77, 78], Furazolidone [78], and Paromomycin [79]. Nitrazoxanide was examined as an alternative oral agents for MTZ-resistant TV but was not found to be very effective [80]. Several combination therapies including TNZ plus ampicillin and multi-dose NTZ [81]. Some plant extracts have shown anti-TV activity, but these have not yet been tested in clinical trials [82].

#### Treatment among HIV-infected women

In an randomized clinical trial (RCT) among HIV-infected women with TV, multi-dose MTZ was found to be superior to single dose treatment [83]. Further analysis revealed that the superiority is only in the presence of bacterial vaginosis (BV) [84]. Studies have also found that antiretroviral therapy may interfere with the efficacy of MTZ among HIV-infected women [85, 86].

It has been estimaged that if CDC recommendation for TV screening and treatment among HIV+ women is followed, that the lifetime cost of new HIV infections prevented would approximate US $159,264,000 via new HIV cases of secondary to female-to-male transmissions prevented [87].

#### Repeat/ persistent infections

**Fig. 1:**
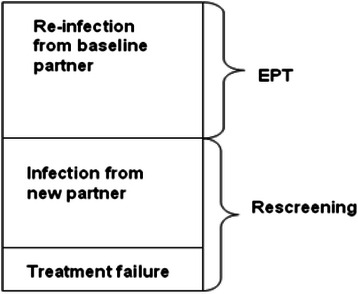
Possible causes of a repeat TV+ test after treatment among TV infected persons

Repeat infections are common, ranging from 5–31 % [88–92], and share similar sequelae to primary infections. While it is clear that the TV repeat infection rate is unacceptably high, the source of these repeat infections is less clear. Possible sources of retest positives after treatment are: re-infection from an untreated/infected baseline partner, infection from a new partner, or treatment failure. Each of these sources of retest positives requires a different approach to prevent ongoing infection (see Fig. 1). For example, if the cause is re-infection, then assuring the original partners are treated (i.e. expedited partner treatment or EPT) is needed. If the source is a new partner or treatment failure, then rescreening is needed.

There have only been a few randomized trials with good follow-up that have compared single dose MTZ to multi-dose. In these trials, cure rates for singe vs. multi-dose MTZ have been shown to be similar (82–88 % vs. 92–94 %) [93, 94]. Both studies found that the single dose had higher rates of side effects (notably nausea and vomiting).

One study that examined the origins of repeat infection found treatment failure to be the most common cause [88]. Potential causes of early repeat TV infections include: drug resistance, non-adherence to treatment, clinical treatment failure, or re-infection from an untreated partner. Single dose therapy has removed adherence as an issue and in vitro resistance testing has consistently demonstrated low rates of resistance. Reported rates of MTZ resistance among mostly non-HIV infected women range from 2.2–9.6 % [89, 95–97] and were usually resolved with repeat MTZ treatment at the same or higher dosage [97]. The most likely sources of repeat infections, therefore, are clinical treatment failure or re-infection from an untreated partner.

In one study of HIV+ and HIV- women, a large proportion of the repeat infections were attributed to treatment failure (i.e. no sexual exposure and no drug resistance) [88]. Resistance appears to play only a minor role in explaining probable treatment failure. TV infected women who were given single dose MTZ and provided with medication to deliver to their sex partner(s), repeat infections rates were high (8 %) and nearly all (92 %) were attributed to clinical treatment failure [88]. Repeat TV infections among HIV+ women are substantially higher with rates between 18.3 and 36.9 % [88, 98, 99] and since these studies used culture, the true rate may be even higher. The molecular mechanism(s) of clinical resistance are poorly understood.

#### Sex partner treatment

Sex partners of patients with TV should be treated. Commonly, patients are told by their providers to tell their partners to seek testing and treatment. This can be problematic because sensitive tests for men are not readily available. Providers may consider treating partners of positive patient presumptively. One method of presumptive partner treatment is called expedited partner therapy (EPT). EPT is the clinical practice of treating the sex partners of patients diagnosed with an STI by providing prescriptions or medications to the patient to take to his/her partner without the health care provider first examining the partner.

One RCT demonstrated that partner treatment with 2 g TNZ resulted in a > 4 fold reduction in repeat infections among TV + index women [100]. Two other studies using 2 g MTZ for male partners of TV infected women found no effect of EPT [90] or a borderline effect [101]. While it is possible that the two studies that used MTZ were either underpowered or did not use the correct control arm, it is also possible, that TNZ is a better treatment for men.

#### Microbiome and TV

There has been recent evidence that TV infection changes or is changed by the microbiome of women [102, 103] and TV treatment is altered by the microbiome [104]. One possible factor in the treatment failure of TV is vaginal flora disturbances. Bacterial vaginosis (BV) is a common vaginal condition in women of childbearing age. The prevalence of BV in the US varies from 29 % in a nationally representative sample (where the prevalence was 3.1 times greater for African-American women compared to whites), 44 % in a group of women at high-risk for HIV [105], and 56 % among injection drug users [106]. Like TV, BV can also increase a woman’s susceptibility to HIV infection [42, 107, 108]. Several studies have shown a strong association between TV and BV [109–112], meaning that the two frequently occur as co-infections among women. While these two vaginal infections have similar symptomatology and are treated with similar medication, the dosing is not the same.

TV has been found to occur more often in the presence of women with a newly identified species of Mycoplamsa called Mnola or Candidatus Mycoplasma girerdii [103, 113]. Brotman et al. found that TV was associated with vaginal microbiota consisting of low proportions of lactobacilli and high proportions of Mycoplasma, Parvimonas, Sneathia, and other anaerobes [114].

In a screening study of HIV-positive women, the prevalence of TV was higher among women who had altered vaginal flora and that the majority (61.0 %) of HIV+/TV+ women also had BV [84]. This high rate of BV that accompanies TV infection among HIV+ women has implications for treatment decisions since multi-dose MTZ is recommended for BV. Martin et al. found that TV prevalence was highest in the women with intermediate Nugent scores confirming the observations of Hillier et al. [115] and Gatski [84]. A heat map analysis of pyrosequencing data showed that the vaginal flora of 18/30 TV+ women had a similar unique microbiota characterized by high abundance of Mycoplasma ssp or Ureaplasma ssp. and relatively low abundance of Lactobaccilus spp. and Gardnerella spp [103], suggesting that TV directly influences microbial environment and confirms the potential importance of interactions between TV and vaginal microbiota.

## Conclusions

TV is an important source of reproductive morbidity and may amplify the acquisition and transmission of HIV and possibly HSV-2. While TV it is the most common non-viral STI globally and it is mostly asymptomatic, it is not a reportable disease and screening programs generally do not exist. High rates of repeat TV positivity after single dose MTZ, the most commonly used treatment regimen, are seen. Since TV appears to be highly susceptible to MTZ in vivo and most repeat TV positivity does not seem to be reinfection, the evidence suggests that single dose MTZ, the most commonly used treatment regimen, is not effective and host factors may be the cause. Scientists should continue to focus on better diagnostic, particularly for men, and treatment for both index persons and their partners and on a better understanding of host and parasite factors that play into treatment failure.
